# *Notes from the Field:* Nontuberculous Mycobacteria Infections After Cosmetic Surgery Procedures in Florida — Nine States, 2022–2023

**DOI:** 10.15585/mmwr.mm7303a4

**Published:** 2024-01-25

**Authors:** Katharine E. Saunders, Juliana M. Reyes, Lorrie Cyril, Molly Mitchell, Stephanie Colter, Jalysa Erskine, Kiara X. McNamara, Jennifer C. Hunter, Kiran M. Perkins, Argentina Charles

**Affiliations:** ^1^Epidemic Intelligence Service, CDC; ^2^Florida Department of Health; ^3^Florida Bureau of Public Health Laboratories; ^4^Division of Healthcare Quality Promotion, National Center for Emerging and Zoonotic Infectious Diseases, CDC.

**Summary**

**What is already known about this topic?**

Nontuberculous mycobacteria (NTM) have been reported as a cause of health care–associated infections.

**What is added by this report?**

Investigation of a case of NTM infection in a patient who received a cosmetic surgical procedure in Florida identified a total of 15 cases in nine states in patients who received cosmetic surgical procedures at the same facility in Florida. Multiple lapses in infection control and prevention were found at an outpatient cosmetic surgery clinic operating with the same staff members.

**What are the implications for public health practice?**

Health care providers should have a high index of suspicion for extrapulmonary NTM when evaluating patients for postsurgical infection after cosmetic procedures and should be aware of the threshold for notifying public health officials of these cases.

## Introduction

*Mycobacterium abscessus* is a species of intrinsically multidrug–resistant, rapidly growing nontuberculous mycobacteria (NTM) known to cause health care–associated infections (*1*) and implicated in skin and soft tissue infections after cosmetic surgical procedures (*2*,*3*). On February 7, 2023, CDC notified the Florida Department of Health (FDOH) of a non-Florida resident with NTM infection after a cosmetic procedure at a surgery clinic (clinic A) in south Florida. FDOH and CDC issued national Epidemic Information Exchange (Epi-X) notices in March 2023 (http://www.cdc.gov/epix) to identify additional infections. During the ensuing investigation in March 2023, FDOH identified a total of 15 NTM infections in patients who underwent surgical procedures by a plastic surgeon in solo practice at clinic A, an alternative practice location that was used during renovation of the permanent facility (clinic B).

## Investigation and Outcomes

### Case Definition

A case was defined as the isolation of *M. abscessus* from a wound culture obtained from a patient who underwent a cosmetic procedure at clinic A during August–December 2022. Among 19 reported infections, a total of 15 patients (including the index patient) from nine U.S. states met the case definition (Table). The other four patients experienced signs and symptoms of postsurgical infection but lacked confirmatory laboratory results and were not included in the analysis. This activity was reviewed by CDC, deemed not research, and was conducted consistent with applicable federal law and CDC policy.*

**TABLE:** Demographic and epidemiologic characteristics of patients with nontuberculous mycobacteria infections after receiving cosmetic surgery at clinic A (N = 15) — Florida, 2022–2023

Characteristic	No. (%)
**Sex**	
Male	0 (—)
Female	15 (100)
**Race or ethnicity**	
Black or African American, non-Hispanic	3 (20)
White, non-Hispanic	1 (7)
White, Hispanic or Latino	2 (13)
Hispanic or Latino	1 (7)
Unknown	8 (53)
**Age group, yrs**	
18–29	4 (27)
30–39	8 (53)
40–49	2 (13)
50–59	1 (7)
**State of residence**	
California	4 (27)
Florida	4 (27)
Illinois	1 (7)
Kansas	1 (7)
Massachusetts	1 (7)
Missouri	1 (7)
Texas	1 (7)
Washington	1 (7)
Wisconsin	1 (7)
**Wound site**	
Buttocks	10 (67)
Abdomen	3 (20)
Hip	1 (7)
Breast	1 (7)
Procedure performed*	
Liposuction	15 (100)
Gluteal augmentation with autologous fat transfer	12 (80)
Abdominoplasty	2 (13)
Breast reduction or lift	1 (7)
More than one procedure	14 (93)
**Outcome**	
Required intravenous antibiotics	6 (40)
Required additional interventions^†^	4 (27)
Inpatient hospitalization^§^	1 (7)
Death	0 (—)

* Patients might have had multiple procedures at the time of surgery; therefore, the total number of procedures exceeds the total number of patients.

^†^ Including computerized tomography–guided percutaneous abscess drainage, needle aspiration, incision and drainage, wound debridement, and surgical skin excision with drainage.

^§^ Data obtained from available records.

### Characteristics of Cases

All patients were women, and the median age was 33 years (range = 24–51 years). The median interval from the procedure to symptom onset was 69 days (range = 33–119 days). Patients reported swelling, purulent drainage, redness, or pain at surgical sites. Pharmaceutical treatments for *M. abscessus *included oral and intravenous antibiotics with prolonged courses up to 2*–*6 months*. *In addition to prescribing medication, health care providers performed incision, drainage, and debridement. (Table). Seven patients’ wound isolates were available for analysis by the Florida Bureau of Public Health Laboratories. Whole genome sequencing determined that four isolates were closely related^†^ (*4*). Clinic A was closed after identification of the cluster; the closure precluded environmental sampling during the investigation.

### On-site Assessment

On March 3, 2023, FDOH conducted an on-site infection control assessment at clinic B, which operated with the same surgeon, staff members, and protocols as did clinic A. The assessment detected gaps in environmental cleaning practices, use of proper personal protective equipment, and disinfection during surgical device reprocessing.

## Preliminary Conclusions and Actions

This cluster was challenging to identify because patients were located throughout the United States and because NTM infections are not nationally notifiable. Collaboration among health jurisdictions and CDC was crucial in identifying the initial extrapulmonary NTM infection. Subsequent case finding required active surveillance to encourage reporting by providers as well as outreach to patients using nontraditional approaches, such as social media and online reviews of businesses (e.g., Facebook or Yelp), because patients reported symptoms on these sites.

### Implications for Public Health Practice

Health care providers should have a high index of suspicion for extrapulmonary NTM when evaluating patients for postsurgical infection after cosmetic procedures and should be aware of the existing threshold for notifying public health officials, realizing NTM infections could develop months after surgery. Standard case definitions and thresholds for reporting to improve surveillance have been published.^§^^,^^¶^ Although the specific source associated with clinic A’s cluster has not yet been identified, FDOH found gaps in infection control, including cleaning practices, use of personal protective equipment, and surgical device disinfection, that can contribute to NTM transmission. FDOH will use these findings to develop additional training for cosmetic surgery clinic staff members statewide to help prevent future outbreaks in this setting (*5*).

## Footnotes

* 45 C.F.R. part 46.102(l)(2), 21 C.F.R. part 56; 42 U.S.C. Sect. 241(d); 5 U.S.C. Sect. 552a; 44 U.S.C. Sect. 3501 et seq.

† Among the seven isolates available for genetic sequencing, all were identified as *Mycobacteroides abscessus*, with six identified as sequence-type 80, and one as sequence-type 422. Four isolates were similar with average nucleotide identity values of 100% and a coding single nucleotide polymorphisms (SNP) difference of 0–3. Florida Bureau of Public Health Laboratories used a 20 SNP cutoff to identify relatedness based on established literature.

§
https://cdn.ymaws.com/www.cste.org/resource/resmgr/publications/Extrapulmonary-NTM-PS_Op-Gui.pdf

¶
https://www.corha.org/wp-content/uploads/2019/09/CORHA-Proposed-NTM-Thresholds-and-Definition-08-19.pdf
